# Anti-angiogenic effects of the thienopyridine SR 25989 *in* *vitro* and *in vivo* in a murine pulmonary metastasis model

**DOI:** 10.1038/sj.bjc.6600142

**Published:** 2002-03-04

**Authors:** M C M Mah-Becherel, J Céraline, G Deplanque, M-P Chenard, J-P Bergerat, J-P Cazenave, C Klein-Soyer

**Affiliations:** Laboratoire de Cancérologie Expérimentale et de Radiobiologie, Institut de Recherche contre les Cancers de l'Appareil Digestif, Hôpitaux Universitaires de Strasbourg, BP 426, 67091 Strasbourg, France; Anatomie pathologie Générale, Hôpitaux Universitaires de Strasbourg, BP 426, 67091 Strasbourg, France; INSERM U. 311, Etablissement Français du Sang-Alsace, 10, rue Spielmann, BP 36, 67065 Strasbourg Cédex, France

**Keywords:** B16 F10 melanoma, metastasis, thienopyridine, SR 25989, anti-angiogenesis

## Abstract

Neovascularisation is a key step in tumour growth and establishment of distant metastases. We have recently demonstrated that the thienopyridine SR 25989 an enantiomer of the anti-aggregant clopidogrel (Plavix®) lacking anti-aggregant activity, inhibits endothelial cell proliferation *in vitro* by increasing the expression of endogenous thrombospondin-1, a natural potent inhibitor of angiogenesis. The anti-angiogenic effect of SR 25989 was further assessed *in vitro* in a quantitative assay of angiogenesis comprising a fragment of rat aorta embedded in a fibrin gel and *in vivo* in a pulmonary metastatic model using C57BL/6 mice inoculated in the foot pad with the highly metastatic melanoma cell line B16 F10. SR 25989 induced a dose dependent inhibition of spontaneous microvessel development *in vitro* reaching half maximal inhibition at around less than 50 μM and caused platelet derived growth factor induced angiogenesis to regress as a function of thienopyridine concentration. *In vivo,* SR 25989 did not alter significantly the growth rate of the primary tumour in the foot pad and did not inhibit development of inguinal nodes which appeared after amputation. However, the number and size of lung metastases were reduced in treated animals when examined at the time of sacrifice. In addition, the few metastases over 1 mm^3^ did not show any neovascularisation, as confirmed by negative von Willebrand immunostaining and in contrast to intense vascularisation seen in metastases developed by control mice. These results confirm that SR 25989 possesses potent anti-angiogenic properties and is able to inhibit metastatic dissemination and growth. The lack of effect on the primary tumour and inguinal nodes illustrates the complexity of the mechanisms involved in tumoural neo-angiogenesis and points out the possibility for distinct processes leading to neovascularisation in primary tumour as opposed to metastases.

*British Journal of Cancer* (2002) **86**, 803–810. DOI: 10.1038/sj/bjc/6600142 www.bjcancer.com

© 2002 Cancer Research UK

## 

For three decades angiogenesis has been recognised as a key step in tumour growth and metastasis development (Folkman, 1995a,b). The great cellular and genetic diversity of solid tumours is responsible for the wide disparity of their responses towards chemical/radiotherapeutic treatments. One common element among their characteristics is the tumour vasculature which shares identical features throughout the tumoral tissues and which is different from the vasculature of normal tissues. The tumour vasculature has an immature endothelium and forms an abnormal network of leaky vessels with chaotic flow (Denekamp *et al*, 1998). Below a critical size of 1–2 mm^3^ metastases are supposed not to need to be vascularised for their nutritive supply (Folkman, 1990). However some tumours can grow above this size without inducing angiogenesis by using some alternative strategies such as vessel cooption (Holash *et al*, 1999) or tumour cells forming their own channels (Barinaga, 1999; Maniotis *et al*, 1999). Despite these particularities, it appears that tumour angiogenesis now represents a promising therapeutic target for the treatment of cancer (Deplanque and Harris, 2000; Kerbel, 2000; Liekens *et al*, 2001). Among the abundance of molecules to which an angiostatic or anti-angiogenic property has been assigned, some of them have today reached the stage of the clinical assay. Although the mechanisms of these molecules are way from being all elucidated, several strategies have been drawn: (i) inhibition of signalling pathways – by targeting tyrosine kinase receptors involving growth factors such as basic fibroblast growth factor (bFGF), platelet derived growth factor (PDGF) and vascular endothelial growth factor (VEGF) which are strong inducers of angiogenesis (Laird *et al*, 2000; Pegram and Slamon, 2000), – by inhibition of the growth factors themselves (Pepper *et al*, 1996; Lee *et al*, 2000), – or by inhibiting adhesion molecule receptors like the integrin α_v_ β_3_ which is essentially expressed in growing capillaries (Brooks *et al*, 1994; Gutheil *et al*, 2000); (ii) inhibition of tumour neovascularisation by means of endogenous polypeptide fragments or proteins having anti-angiogenic or angiostatic properties: molecules such as angiostatin, an internal fragment of plasminogen (O'Reilly *et al*, 1994), endostatin, a fragment of collagen XVIII (O'Reilly *et al*, 1997), platelet factor 4 (PF4), (Maione *et al*, 1990), interferons (Ezekowitz *et al*, 1992) and thrombospondin-1 (TSP-1), a matricellular protein (Bornstein, 1995) appear among these molecules (reviewed in Sage, 1997; Carmeliet and Jain, 2000; Kerbel, 2000); (iii) reassessment of molecules which were not originally developed as anti-tumour angiogenesis drugs but for which anti-angiogenic properties were ‘accidentally’ discovered. Thalidomide, a synthetic sedative, microtubule-affecting drugs such as taxanes or low doses of methotrexate, are among this type of ‘accidental’ anti-angiogenic molecules presently tested in clinical assays as adjuvants of anti-tumour therapy (reviewed in Kerbel *et al*, 2000); (iv) finally one possible strategy could consist in inducing the expression of constitutive endogenous anti-angiogenic molecules.

We have recently demonstrated that a molecule belonging to the thienopyridine family, SR 25989 inhibits constitutive and aFGF or bFGF driven endothelial cell proliferation and migration in a wound healing model (Klein-Soyer *et al*, 1994). The decrease in cell proliferation is correlated to an upregulation of the expression of TSP-1 (Klein-Soyer *et al*, 1997). Furthermore, preliminary experiments using fibrin gel chambers implanted in the dorsal subcutaneous space of the rat demonstrated that SR 25989 inhibits neovascularisation *in vivo* (Cazenave and Herbert, 1992; Toti *et al*, 2001). Consequently, these antiangiogenic effects of SR 25989 were further assessed and we report presently the results obtained both *in vitro* in a quantitative assay of angiogenesis using the model developed by Nicosia and Ottinetti (1990) and *in vivo* in a murine pulmonary metastatis model using C57BL/6 mice inoculated with the highly metastatic melanoma cell line B16 F10.

## MATERIALS AND METHODS

### Animals, cells and materials

Male Wistar rats weighing between 200 and 250 g and male mice C57BL/6 weighing between 22 and 25 g, both kinds pathogen free, were obtained from Charles River (St Aubin, Les Elbeufs, France). Animal care and experimentation were in accordance with the institutional guidelines from the Ministère Français de l'Agriculture and The UKCCCR Guidelines (1998) for the welfare of animals in experimental neoplasia. All animal were fed a diet of animal chow and water *ad libitum*. The animals were anaesthetised with intramuscular xylasin (8 mg kg^−1^) (Rompun®, Bayer Leverkusen, Germany) and ketamin (40 mg kg^−1^) (Ketalar®, Substantia Division Santé, Courbevoie, France). The highly metastatic B16 F10 mouse melanoma cell line (Fidler, 1973) was a generous gift from Dr IJ Fidler. Agarose type VI A, rat fibrinogen (fraction I, >90% clottable), ε-aminocaproic acid, magnesium sulphate (MgSO_4_, cell culture tested) and calcium chloride (CaCl_2_) were from Sigma (St Louis, MO, USA). Cell culture medium (DMEM/HAM F12, with 15 mM HEPES; RPMI 1640) Hank's balanced salt solution, L-glutamine, antibiotics (penicillin, streptomycin), fungizone and foetal calf serum (myoclone plus, virus and mycoplasma screened) (FCS) were from Gibco (Paisley, UK). Petri culture dishes, six well dishes and culture flasks (tissue culture grade) were from Falcon, Becton Dickinson Company (Lincoln Park, NJ, USA). Human serum albumin (HSA) solution was from the Etablissement de Transfusion Sanguine de Strasbourg, France. Human α-thrombin was prepared in the laboratory according to published methods (Ngai and Chang, 1991) and platelet derived growth factor (PDGF) was from R&D systems (Minneapolis, MN, USA). Recombinant hirudin and the thienopyridine, SR 25989, were a gift from Sanofi Recherche (Toulouse, France). Anti-human von Willebrand factor (vWf) antibody, immunoglobulin fraction, and monoclonal mouse anti-human α-smooth muscle actin (α-SMA) antibody (clone1A4; IgG2aκ) were from Dako A/S, (Glostrub, Denmark). Polyclonal rabbit immunoglobulin fraction was prepared from non-immunised rabbits. Purified mouse IgG2a (κ chain) (MOPC-173) control immunoglobulins were obtained from Pharmingen (San Diego, CA, USA). Eukitt® mounting solution was from Poly Labo Company (Strasbourg, France). All other chemicals were of analytical grade and from Sigma Immuno Chemicals (Sigma-Aldrich Corp., St Louis, MO, USA) or Merck (Darmstadt, Germany).

### Cell culture and proliferation assay

B16 F10 cells were routinely grown in RPMI 1640, 2 mM glutamine, 100 U ml^−1^ penicillin, 100 μg ml^−1^ streptomycin, and 10% FCS unless otherwise stated. Exponentially growing cells were sparsly seeded (5000 cells per cm^2^) in 96-multiwell plates in the presence of 10% FCS. After 24 h when cell adhesion was achieved, the medium was changed and the serum concentration set to 10, 5 or 2% FCS. SR 25989 was added at 37.5 and 150 μM. These two concentrations were chosen as previous works have shown that SR 25989 up to 37.5 μM had no effect on the proliferation of human endothelial cells or skin fibroblasts but displayed significant inhibitory activity in the range of 75–150 μM (Klein-Soyer *et al*, 1994, 1997). The medium was further changed at days 4 and 7 in identical conditions and the drug was added at each medium change. At days 4, 7 and 10 representative samples were fixed in 2% paraformaldehyde solution and B16 F10 proliferation was estimated by quantification of crystal violet staining of the cells with a microplate reader (Gillies *et al*, 1986).

### Clonogenic assay

The effects of SR 25989 on cell survival was investigated in a clonogenic assay as follows: exponentially growing B16 F10 cells were used for the experiments. Single cell suspensions were prepared from B16 F10 cultures which had been submitted to increasing concentrations of SR 25989 (0 to 400 μM) for 4 h in medium containing 10% FCS. The suspensions were serially diluted in medium containing 10% FCS and the different B16 F10 cell dilutions were seeded in triplicate in 10 mm Petri dishes. After 7 to 10 days incubation without medium exchange, the cultures were carefully rinsed with PBS and stained with Giemsa dye and colonies containing more than 50 cells (Brown and Wouters, 1999) were counted to establish the survival curve.

### Quantitative assay of angiogenesis *in vitro*

*In vitro* angiogenesis experiments were performed using the model described by Nicosia and Ottinetti (1990) with slight modifications. Male Wistar rats were anaesthetised with rompun/ketalar solution. Prior to dissection of the thoracic aorta a bolus of hirudin (3.5 mg kg^−1^ i.v.) was injected into the animal to inhibit any trace of generated thrombin. The vessel was placed in survey medium consisting of Hank's balanced salt solution containing 1% HSA, 100 U ml^−1^ penicillin, 100 μg ml^−1^ streptomycin, 0.25 μg ml^−1^ fungizone. It was processed within 2 h following collection. The aorta was carefully cleaned from surrounding fibroadipose tissue and extensively washed in survey medium containing 2.5 times concentrated antibiotics and fungizone. Angiogenesis chambers consisted of agarose rings with 11 mm inner diameter. Each agarose ring was placed in one well from a six well dish. One millimetre long aorta fragments were embedded in fibrin gels according to published procedures (Nicosia and Ottinetti, 1990) as follows: the fibrinogen solution (3 mg ml^−1^ in PBS without Ca and Mg) was allowed to clot by adding α-thrombin (0.2 NIH U ml^−1^ in 3 mM CaCl_2_ solution) the bottom of each agarose well was coated with 100 μl of clotting fibrinogen solution and the fibrin gel was allowed to polymerise at 37°C. Then, the aorta rings were transferred to the wells and positioned on top of the gelling solution. The agarose wells was then filled with fibrinogen clotting solution. After polymerisation at 37°C, serum-free culture medium consisting in DMEM/HAM F12 supplemented with MgSO4 (10 mM final), 100 U ml^−1^ penicillin, 100 μg ml^−1^ streptomycin and 2.5 μg ml^−1^ fungizone, was added to each dish. In order to prevent fibrinolysis, ε-amino caproïc acid was added throughout the experiments. The medium was changed every second day and enumeration of newly formed vessels, arising from the intimal endothelium and the vasa vasorum of the aorta rings, was performed every day for 8 to 10 days on an inverted microscope (magnification ×40, Nikon Diaphot TMD). Experiments in which occasionally smooth muscle cells or fibroblasts developed, recognisable because they formed cell layers instead of tubules, were systematically discarded.

### Effect of the thienopyridine SR 25989 on spontaneous angiogenesis and angiogenesis induced by PDGF *in vitro*

SR 25989 in the range 1.5 to 150 μM was added to the culture medium, starting from day zero and at every medium change. The formation of neovessels was quantified according to criteria described by Nicosia and Ottinetti (1990). In a second set of experiments PDGF (1 ng ml^−1^) was added from time zero and every second day until day 4. This growth factor presented to be the most potent to induce angiogenesis in this model as compared to bFGF (data not shown). Also, it had been shown previously that ticlopidine, another member of the thienopyridine family inhibits the release of PDGF from platelets (Morimoto *et al*, 1992). Due to the difference in reactivity between individual animals the number of spontaneously growing microvessels was heterogeneous between separate experiments. Nevertheless, for a given experiment this concentration of the growth factor was shown to enhance spontaneous new capillary formation by up to 5–10 times over control values in our system (unpublished observations). At day 4, when angiogenesis was well established, increasing concentrations of SR 25989 were added once to culture medium and neovascularization was quantified 24 h thereafter.

### Experimental spontaneous lung metastasis model

Exponentially growing B16 F10 cells detached by brief exposure to 0.05% Trypsin/0.02% EDTA were washed twice in complete medium and finally resuspended in Hank's balanced salt solution. A single suspension of cells (depending on the experiment, five to 8.5×10^5^ cells, in 100 μl) was injected into the foot pad of C57BL/6 mice. This model was chosen in order to observe the effect of the drug on the development of the primary tumour and on the grounds of the high vascularisation of the foot pad. This makes this model closer to the ‘artificial pulmonary metastasis model’ which requires injection of the tumour cells directly in the tail vein than to the model implying implantation of tumour cells in the dorsal subcutaneous space. The injection schedule systematically alternated between mice in the control group and mice in the SR 25989 treated group to minimise differences which might be attributed to the duration of the injection protocole. At the end of the inoculation procedure the viability of B16 F10 cells assessed by Trypan blue dye exclusion was ≈85%. Animals were given either SR 25989 50 mg kg^−1^ (equivalent to 120 μmoles kg^−1^) or normal saline (control groups) intraperitoneally daily from the day of tumour inoculation to the time of sacrifice. The animals were alert throughout the experiment, did slowly gain weight during primary tumour development and significantly after amputation. The tumour bearing foot was amputated at day 23. At this time the mean tumour size reached 500 mm^3^. At distance from amputation one mouse from each series was sacrificed in order to assess the presence of metastases. This allowed to determine the time of sacrifice and autopsia before the development of metastases would cause dyspnoea. The mice were sacrificed around day 40. The tumours and inguinal lymph nodes were measured with a dial- caliper and the volumes calculated using the following formula: width^2^×length×0.52. The number and the size of metastatic foci on the pulmonary surface were macroscopically quantified. The primary tumour, inguinal lymph nodes and lungs were fixed in 10% formalin solution and embedded in paraffin.

### Immunohistochemistry

Paraffin-embedded tumours, inguinal lymph nodes and lung sections were stained for vWf and for α-SMA by standard indirect immunoperoxidase staining. Proteolytic predigestion of the formalin fixed tissue with 0.05% Trypsin for 30 min at room temperature was performed prior to incubation with anti-vWf antibodies. Primary antibodies were diluted according to the manufacturer's recommendations and the tissue sections were incubated overnight at 4°C. Control staining was carried out in each case by replacing the primary antibody by matched isotypic non immune immunoglobulins. Next, the samples were incubated with the appropriate peroxidase conjugated secondary antibodies for 1 h at room temperature. Tissue labelling was revealed by 3,3′-diaminobenzidine staining, and the samples finally counterstained with haematoxylin (Harris formula) and mounted in Eukitt®.

### Statistical analysis

The effects SR 25989 were compared by variance analysis followed by the Neuman–Keuls test, using the statistical software STAT-ITCF (ITCF, Boigneville, France).

## RESULTS

### Inhibition of spontaneous angiogenesis *in vitro* by the thienopyridine SR 25989

**Figure 1:**
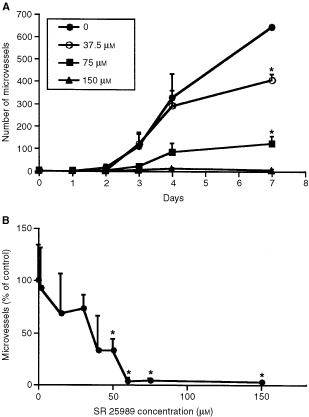
Inhibition of angiogenesis *in vitro* by SR 25989. (**A**) Kinetics of microvessels development as a function of SR 25989 concentration. Results are the mean±s.e.m. of four independent experiments performed in triplicates. (**B**) Number of microvessels as a function of increasing concentrations of SR 25989. Results (expressed as a percentage of control values) are the mean±s.e.m. of eight independent experiments with three angiogenesis chambers per condition. *Significantly different from control (*P*<0.05).

The thienopyridine was added to angiogenesis chambers in serum-free medium at the following concentrations: 37.5, 75 and 150 μM, starting from day zero and at each medium change. As already published (Nicosia and Ottinetti, 1990), the fibrin gel stimulated an angiogenic process and microvascular sprouts developed with time. SR 25989 inhibited the formation of new vessels in a dose dependent manner and inhibition was almost complete for the higher concentration (Figure 1a

). Evaluation of angiogenesis at day 7 as a function of SR 25989 concentration showed that half maximal inhibition was reached at less than 50 μM and was almost complete for concentrations over 60 μM SR 25989 (Figure 1b).

### Effects of SR 25989 on angiogenesis *in vitro* induced by PDGF

**Figure 2:**
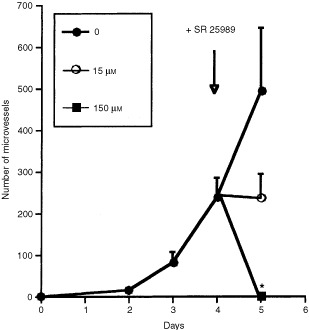
Effect of SR 25989 on angiogenesis *in vitro* induced by PDGF. PDGF (1 ng ml^−1^) was added to angiogenesis chambers until day 4. Then increasing concentrations of SR 25989 were added once (arrow). Microvessel formation was evaluated every day. Results are the mean±s.e.m. of three separate experiments with three angiogenesis chambers per condition. *Significantly different from control (*P*<0.05).

PDGF (1 ng ml^−1^) was added starting from time zero of the experiment. This concentration of the growth factor enhanced the formation of spontaneous capillaries by 5 to 10 times over control values in a given experiment. When neovascularization was well established, at day 4, a low and a high concentration of SR 25989 were added to the culture medium and angiogenesis chambers incubated for one more day. When compared to the controls devoid of thienopyridine, the low concentration of SR 25989 (15 μM) inhibited further increase of newly formed microvessels (Figure 2

) while the high concentration (150 μM) SR 25989 caused a total regression of the already established angiogenesis with a complete disappearance of the capillaries, leaving only dead isolated cells.

### Inhibition of B16 F10 cells proliferation by SR 25989

**Figure 3:**
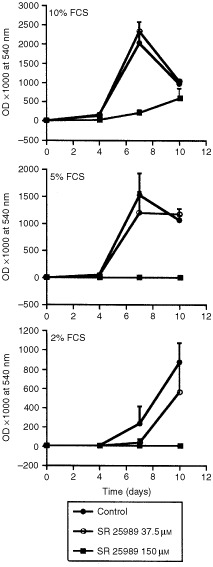
B16 F10 cells proliferation in the presence of SR 25989. B16 F10 cells were grown in the presence of 37.5 or 150 μM SR 25989 in medium containing 2, 5 or 10% FCS and proliferation estimated by colouri metric quantification after Cristal Violet staining as described in Materials and Methods. Results are the mean±s.e.m. of four independent experiments performed in duplicates.

The serum induced proliferation of B16 F10 cells was analysed in the presence of SR 25989 used at two doses encompassing the range of concentrations used in the preceding experiments. Control B16 F10 cells, as quantified by crystal violet staining of adherent cells, proliferated exponentially up to day 7 in the presence of 5 and 10% FCS and up to day 10 with 2% FCS (Figure 3

). The apparent decrease in cell density observed at day 10 in the presence of 5 and 10% FCS was due to cell detachment caused by extremely high cell density. As previously shown for human endothelial cells and skin fibroblasts (Klein-Soyer *et al*, 1994, 1997) the lower concentration (37.5 μM) did not significantly affect B16 F10 cell proliferation as compared to corresponding control cultures. In contrast, the higher concentration (150 μM) totally inhibited the proliferation of the cells cultivated in 2 and 5% FCS and inhibited by over 90% the proliferation of B16 F10 cells cultivated in 10% FCS demonstrating cytostatic effects of this molecule *in vitro* (Figure 3).

### Effects of SR 25989 on B16 F10 cells survival

**Figure 4:**
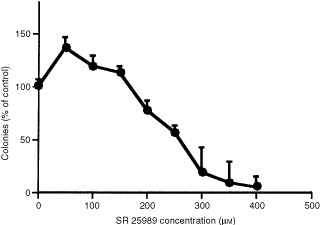
Clonogenic survival of B16 F10 cells treated with SR 25989. B16 F10 cells were exposed to increasing concentrations of the thieno pyridine as described in Materials and Methods. Colonies containing more than 50 cells were counted at day 8. Mean±s.e.m. of one representative experiment out of three performed in triplicates.

In order to verify that SR 25989 should not have major toxic effects for cells in the range of concentration used for proliferation assays, the clonogenic survival of B16 F10 cells was analysed after exposure for 4 h to increasing concentrations of SR 25989 (50 to 400 μM). Up to 150 μM, SR 25989 did not decrease B16 F10 cells survival. For 200 μM the clonogenicity was only reduced by 20% thereafter it decreased rapidly and dose dependently reaching less than 5% for 400 μM (Figure 4

).

### SR 25989 does not affect the development of primary tumours and inguinal lymph nodes

When B16 F10 cells were inoculated into the foot pad of C57BL/6 mice melanic marks appeared as soon as day 7 after injection. Primary tumours developed equally in control and treated animals and the tumour bearing foot was amputated when tumours reached a mean volume of 500 mm^3^. Although tumours from the series treated with SR 25989 tended to develop at a lower rate a few days prior to amputation the average volume attained at this time was not significantly smaller than in the control series (data not shown). The overall vascularisation (macro and microvascularisation) as assessed by vWF and α-SMA staining, was not different between the two series. The extent of necrotic areas, estimated on Hematoxylin-Eosin stained sections seemed however to be slightly larger in treated series although this difference did not appear to be statistically significant (data not shown). From the time of amputation most of animals developed inguinal lymphadenopathies at the site of the tumour bearing foot. The frequency of appearance of lymph nodes was similar between control and treated animals (75.8±13.9% *versus* 64.8±13.2%, mean±s.d., *n*=3) also the volumes of the lymph nodes were not different (data not shown). The health state of the mice was identical in control and SR 25989 treated mice although the weight gain was slightly but not significantly lower in the latter group. Again no significant histological difference could be observed between the two series (data not shown).

### Inhibition of the development of spontaneous pulmonary metastasis by subcutaneously administered SR 25989

**Figure 5:**
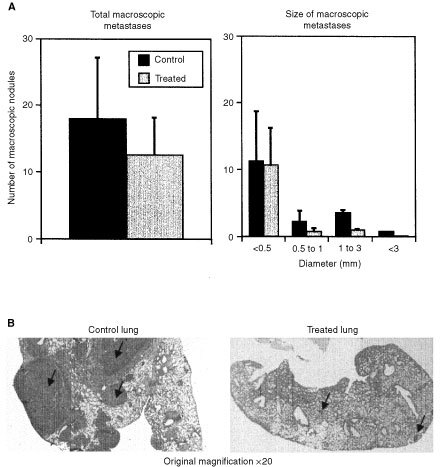
Inhibition of the development of pulmonary metastases by SR 25989. (**A**) The number and the size of macroscopic pulmonary metastases were quantified at autopsia. Histogram left represents the mean of total maroscopic metastases. Histogram right represents the distribution of the metastases as a function of their diameter. Results are the mean±s.e.m. for 11 control mice and 10 treated mice. One representative experiment out of three. (**B**) Paraffin embedded lung sections, Hematoxylin-Eosin staining. Left : control lung showing numerous coalescent metastases. Right: treated lung showing small peripheral or intrapulmonary metastases.

**Figure 6:**
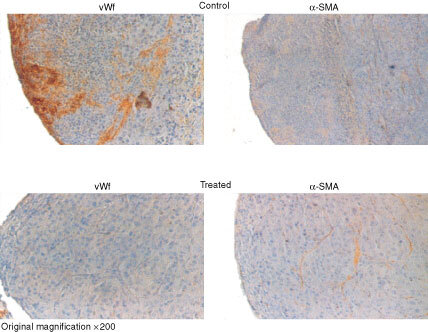
Representative immunohistochemistry of pulmonary metastases from control and SR 25989 treated mice. Paraffin embedded lung sections were labelled with anti-von Willebrand factor antibody (vWf) or anti-α-Smooth muscle actin antibody (α-SMA) as described in Materials and Methods. An intense labelling for vWf can be seen in a metastase from a control lung (upper left), this labelling is totally absent in a metastase of identical size from a treated lung (lower left). α-SMA labelling in the metastases from adjacent sections of the lungs from control and treated mice (right).

The average number of macroscopic pulmonary metastatic foci was diminished by 33% in animals treated with SR 25989 (Figure 5A

left) when examined at the time of sacrifice. In addition when the metastases were distributed as a function of their diameter, it appeared that the size of the metastases was significantly greater in control animals and that most of the metastases from SR 25989 treated animals were classified in the category of smallest size (Figure 5A right). Histological sections of lungs from control mice showed large confluent metastases invading the entire pulmonary tissue and emerging at the surface of the lung while for SR 25989 treated mice the metastases remained significantly smaller and mainly localised at the pulmonary surface (Figure 5B). Immunostaining with a specific antibody to α-SMA demonstrated that most of the metastases initially formed cuffs around preexisting vessels or obstructed vascular lumens (data not shown). Angiogenesis was analysed using antibodies to vWf, the specific marker of endothelial cells. Normal preexisting vessels in the lung were stained but neovascularisation was absent from small (<2 mm diameter) metastases in both control and SR 25989 treated mice. In larger metastases numerous new capillary sprouts essentially located at the periphery of metastases were found in controls but were totally absent in metastases of similar size from SR 25989 treated mice (Figure 6

left). Mature vascularisation visualised by α-SMA staining appeared otherwise similar in the metastases from control and SR 25989 treated animals (Figure 6 right).

## DISCUSSION

The present results demonstrate that SR 25989, a member of the thienopyridine family, which has previously been shown to display anti-angiogenic properties *in vitro* (Klein-Soyer *et al*, 1994, 1997) also inhibits the development of metastases through an anti-angiogenic process in a murine pulmonary metastasis model.

Despite the availability of an ever-increasing number of potential tumoral prognostic markers and the use of aggressive therapies the prognosis of many cancer pathologies has to date only little been improved. This underlines the crucial need for defining new specific criteria integrating the vast diversity of the tumoral genetic alterations. One such strategy has since recent years been approached through the means of angiogenesis for several reasons: (i) the passage from normal tissue to hyperplasia and to tumorigenesis is related to an angiogenic switch (Hanahan and Folkman, 1996); (ii) angiogenesis is a dynamic balance between stimulators and inhibitors (Hanahan and Folkman, 1996; Iruela-Arispe and Dvorak, 1997). In the absence of angiogenesis the balance is in favour of the inhibitors which as such can account for tumour and micrometastases ‘dormancy’ (Folkman, 1995a; Holmgren *et al*, 1995); (iii) Due to its specific characteristics, the tumour vasculature constitutes a common feature between the various tumoral tissues (Denekamp *et al*, 1998). These data have opened an extensive field of investigation to find new anti-angiogenic molecules. Among these molecules the thienopyridine ticlopidine has been shown to reduce the progression of non proliferative diabetic retinopathy (TIMAD Study Group, 1990; Guillausseau, 1994) and to inhibit pulmonary metastasis development in mice (Kohga *et al*, 1981; Bando *et al*, 1984). These results prompted us to evaluate the potential anti-angiogenic properties of another member of the thienopyridine family SR 25989 which is an enantiomer of the anti-aggregant clopidogrel (Plavix) but which lacks anti-aggregant activity.

We have previously shown that SR 25989 inhibited spontaneous and growth factor induced endothelial cell migration and proliferation in a mechanical wound repair model (Klein-Soyer *et al*, 1994). Now, the quantitative angiogenesis assay (Nicosia and Ottinetti, 1990) allowed to further assess the anti-angiogenic properties of the thienopyridine *in vitro*: (i) SR 25989 inhibited in a dose dependent manner the spontaneous microvessel formation from rat aorta fragments embedded in a fibrin gel; (ii) It caused the regression of microvessel development induced by the growth factor PDGF. Hence, we went on evaluating the anti-angiogenic effects of this thienopyridine in a murine pulmonary metastasis model.

High concentrations of SR 25989 displayed cytotoxic effects on the proliferation of B16 F10 cells *in vitro,* however it apparantly did not significantly affect the development of the primary tumour at the therapeutic doses assayed in mice as far as the size of the tumour was considered. For principles of animal care and comfort it was not possible to fully analyse the slight inhibitory effect observed on SR 25989 treated tumour right before amputation. That SR 25989, which potentially inhibits angiogenesis, did not alter tumour growth and lymph node development can be explained by the high vascularisation of the foot pad. This site of injection has been chosen on this purpose to favour tumour cell shedding in the circulation thus allowing distant metastasis development. The already existing vascularisation may be sufficient to explain the regular growth of primary tumours and development of proximal lymph nodes perhaps by vessel cooption (Holash *et al*, 1999). Alternatively, considering the initially high number of tumour cells injected in the foot pad and subsequently isolated tumour cells shed in the circulation, the balance between angiogenesis activators and inhibitors may have been different in the primary tumour and in developing metastases. Thus the available concentration of SR 25989 could have been insufficient to efficiently inhibit angiogenesis activators participating to primary tumour development. Although the thienopyridine did not significantly hinder primary tumour growth, a striking effect of SR 25989 could be observed on the development of pulmonary metastases. Both the number and the size of the metastases were reduced in animals treated with the thienopyridine. While in lungs from control animals numerous coalescent metastases were observed, the lungs from SR 25989 treated animals contained only few metastases and these were significantly smaller. In large metastases from control mice immature vascularisation could be vizualized by vWf immunostaining. This type of labelling was always absent in lung sections from SR 25989 treated animal. This reinforces the fact that SR 25989 inhibits distant metastasis development through an anti-angiogenic process. Such a paradoxical effect in which an angiogenesis inhibitor has no effect on primary tumour growth but a dramatic effect on metastases development seems not to be seldom and has also very recently been reported for the α_v_β_3_ integrin antagonist S-247 (Griggs *et al*, 2001). It has been shown in preliminary experiments that 50 mg kg day^−1^ of SR 25989 given orally to the rat using the model of fibrin gel chambers implanted in the subcutaneous dorsal space of the rat as described by Dvorak *et al* (1987), that SR 25989 significantly inhibits the development of capillaries within the fibrin gel (Cazenave and Herbert, 1992; Toti *et al*, 2001). The dose of SR 25989 that we used here is within the range used for other angiogenesis inhibitors such as TNP-470 (30 mg kg day^−1^) (Gervaz *et al*, 2000) AG3340 (100 mg kg^−1^ daily) (Shalinsky *et al*, 1999), ZD6474 (7.5 to 30 mg kg day^−1^) (Wedge *et al*, 2001) or angiostatin and endostatin (20 mg kg^−1^ daily) (Lush *et al*, 1999). It did not otherwise significantly alter the health and the survival of the mice nor induce any specific secondary effect as compared to control mice treated with normal saline.

Previous results from our laboratory suggest that the anti-angiogenic effects of SR 25989 could possibly be related to an upregulation of endogenous TSP-1 (Klein-Soyer *et al*, 1997). This matricellular glycoprotein is a complex protein displaying multiple functions mediated by interactions through identified domains with a wide range of matrix proteins and cell surface receptors (Bornstein, 1995). TSP-1 regulates tumour growth and metastasis development through an anti-angiogenic process (Roberts, 1996; DiPietro, 1997). Its involvement in anti-angiogenic processes seems to proceed through different mechanisms in particular by inducing endothelial apoptosis through a caspase pathway (Nor *et al*, 2000) or by acting as a ‘molecular bridge’ in adhesion complexes involving integrins such as α_v_ β_3_ (Savill *et al*, 1992; Luscinskas and Lawler, 1994) thus reducing the adhesive contacts between cells and extracellular matrix (Sage and Bornstein, 1991). Recently, an upregulation of TSP-1 has been observed in murine mammary carcinoma after intratumoral injection of endostatin plasmid, which itself is known as an angiogenesis inhibitor (Ding *et al*, 2001). For all these reasons TSP-1 appears now among the most relevant endogenous inhibitors of angiogenesis.

The thienopyridine SR 25989 inhibits metastatic dissemination and growth through an anti-angiogenic process possibly by up-regulating the endogenous angiogenesis inhibitor TSP-1. SR 25989 seems to show valuable anti-angiogenic effects both *in vitro* and *in vivo* and thus deserves further evaluation in view of its potential use as an adjuvant treatment of cancer therapy in humans.
